# Structural Phase Transition and In-Situ Energy Storage Pathway in Nonpolar Materials: A Review

**DOI:** 10.3390/ma14247854

**Published:** 2021-12-18

**Authors:** Xian-Kui Wei, Rafal E. Dunin-Borkowski, Joachim Mayer

**Affiliations:** 1 Ernst Ruska-Centre for Microscopy and Spectroscopy with Electrons, Research Centre Jülich, 52425 Jülich, Germany; rdb@fz-juelich.de (R.E.D.-B.); mayer@gfe.rwth-aachen.de (J.M.); 2 Gemeinschaftslabor für Elektronenmikroskopie (GFE), RWTH Aachen University, 52074 Aachen, Germany

**Keywords:** energy storage, in situ, antiferrodistortive-to-ferrodistortive, phase transition, metal-to-insulator transition, ionic migration

## Abstract

Benefitting from exceptional energy storage performance, dielectric-based capacitors are playing increasingly important roles in advanced electronics and high-power electrical systems. Nevertheless, a series of unresolved structural puzzles represent obstacles to further improving the energy storage performance. Compared with ferroelectrics and linear dielectrics, antiferroelectric materials have unique advantages in unlocking these puzzles due to the inherent coupling of structural transitions with the energy storage process. In this review, we summarize the most recent studies about in-situ structural phase transitions in PbZrO_3_-based and NaNbO_3_-based systems. In the context of the ultrahigh energy storage density of SrTiO_3_-based capacitors, we highlight the necessity of extending the concept of antiferroelectric-to-ferroelectric (AFE-to-FE) transition to broader antiferrodistortive-to-ferrodistortive (AFD-to-FD) transition for materials that are simultaneously ferroelastic. Combining discussion of the factors driving ferroelectricity, electric-field-driven metal-to-insulator transition in a (La_1−x_Sr_x_)MnO_3_ electrode is emphasized to determine the role of ionic migration in improving the storage performance. We believe that this review, aiming at depicting a clearer structure–property relationship, will be of benefit for researchers who wish to carry out cutting-edge structure and energy storage exploration.

## 1. Introduction

**Figure 1:**
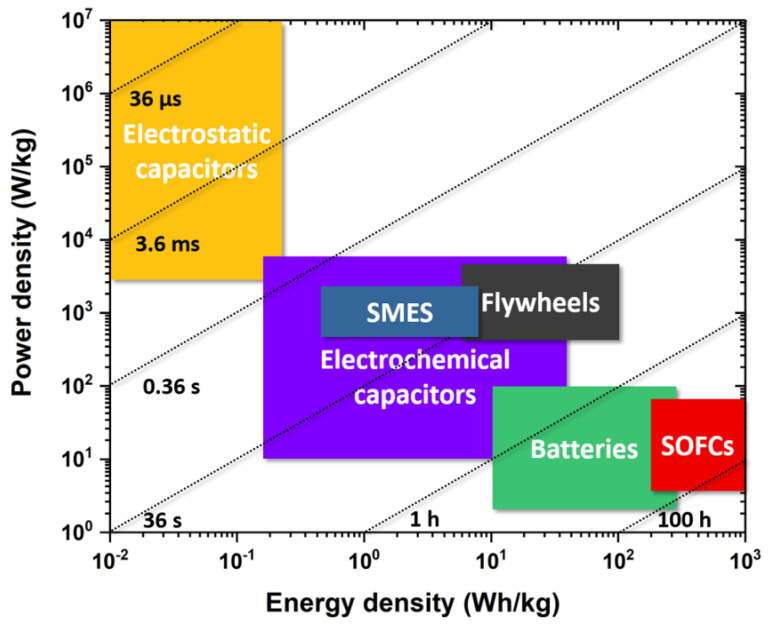
Log-scale Ragone plot showing a performance comparison between different energy storage devices, including superconducting magnetic energy storage (SMES). Conceptually, the energy density (Wh/kg, vertical axis) describes how much energy is available, while the power density (W/kg, horizontal axis) shows how quickly the energy can be delivered. The sloping lines indicate the required time to get the charge in or out of a device [40].

Renewable energies harvested from solar, wind and chemical fuels are playing ever-greater roles in our lives [1,2]. However, their widespread utilization is largely impeded by the underdeveloped energy storage technologies. Thus far, popular electrical energy storage systems consist of the following categories: (1) solid oxide fuel cells (SOFCs), (2) batteries, (3) electrochemical capacitors and (4) dielectric capacitors. From the Ragone plot, one can see that the SOFCs have the highest energy density, while the electrostatic capacitors possess the highest power density, which is up to 10^7^ W/kg. In between, the electrochemical capacitors show a tendency of partially replacing batteries due to their fast-growing energy density; see Figure 1. Specifically, the ultrafast charging/discharging rates, at microsecond level, make dielectric capacitors widely used in devices such as motor starters, high-power lasers, signal processing and sensors [3]. In retrospect, the technological advancement may date back to the ferroelectric (FE) phenomena of Rochelle salt found by Valasek in 1921 [4,5]. During World War II [6], the discovery of BaTiO_3_ and its usage in high-energy-density capacitors launched a research boom in the field of FE materials. Possibly enlightened by the whimsical concept of antimatter introduced by Schuster in 1898 [7], Néel and Kittel proposed the concepts of antiferromagnet and antiferroelectric (AFE) in 1936 and 1951, respectively, to explain magnetic and dielectric anomalies at Curie temperature (*T_C_*) [8,9].

Although receiving less attention than FEs, AFE materials have been investigated for their intriguing physical properties, including the origin of antiferroelectricity [10,11,12,13], electromechanical coupling [14], electrocaloric effect [15,16,17] and negative capacitance [18]. In fact, AFE-based materials have been proposed to design memory devices [19,20]. In recent years, both AFE-based and relaxor FE (RFE)-based capacitors [21,22,23,24,25,26] have been widely investigated due to their reducible energy loss [22,27], improvable maximum polarization (*P_max_*), breakdown electric field (*E_B_*) [28,29,30] and energy efficiency (*η*) [31,32]. Recently, a series of promising data have been reported [32,33,34,35,36]. Using a superparaelectric design, Pan et al. achieved very high recoverable energy density (*J_rec_* = 152 J/cm^3^) in BiFeO_3_-BaTiO_3_-based RFE thin films [21,37,38]. By means of Sr doping, Acharya et al. reported ultrahigh energy efficiency (*η* = 97%) in AFE Pb_1-x_Sr_x_HfO_3_ thin films [39], where the *J_rec_* and *E_B_* were 77 J/cm^3^ and 5.12 MV/cm, respectively. More surprisingly, Hou et al. found the highest energy density reported thus far (*J_rec_* = 307 J/cm^3^) in SrTiO_3_/La_1-x_Sr_x_MnO_3_ (STO/LSMO, x ≈ 1/3) thin films, which have values of *P_max_* ≈ 125 μC/cm^2^, efficiency *η* ≈ 89% and *E_B_* ≈ 6.8 MV/cm [28]. For representative dielectric films and bulks, a comparison of their energy storage performance is shown in Table 1.

Given a “mismatched” structure–property relationship, one may naturally ask: how does a nonpolar SrTiO_3_ deliver such a high *P_max_* value and energy storage density? Although static structural and compositional characterizations are very helpful, in situ phase transition and storage pathway studies are more promising in unveiling the hidden mysteries. Compared with macroscopic property investigation, in situ dynamic structure study lags far behind in the field of dielectric capacitors. An important reason lies in the fact that there exists a large mismatch between the structural response time for energy storage [47] and the data collection time [48,49,50]. For the former, this is usually completed at the millisecond scale or within an even shorter time. Meanwhile, for the latter, e.g., X-ray diffraction (XRD) and selected area electron diffraction (SAED), they usually take several seconds or even minutes in collecting a dataset. In comparison with RFEs [21,23,51], AFEs are more widely investigated in structural phase transitions under in-situ conditions. The reason resides in the fact that the AFE-to-FE transitions inherently couple with the energy storage process [32,52,53,54].

Building on the established phase transition framework, this review seeks to broaden the research scope from AFEs to nonpolar materials, which are simultaneously ferroelastic or antiferroelastic, in the quest for promising energy storage materials. Under in situ conditions, this entails the extension of well-known AFE-to-FE transition to broader antiferrodistortive-to-ferrodistortive (AFD-to-FD) transition. By summarizing external field-driven phase transitions, research progress regarding in situ and atomic-scale structural characterization, achieved by using direct light-element imaging, is highlighted to understand the energy storage mechanism. Associated with ionic migration across the dielectric/electrode interface, we further discuss the electric field-driven metal-to-insulator (M-I) transition in electrodes and its potential impact on tuning the macroscopic energy storage density of dielectric capacitors.

## 2. Fundamentals of Capacitor Energy Storage

A dielectric capacitor is constructed in a parallel-plate form, i.e., a dielectric layer sandwiched by two conductive electrodes. The physical quantity that manifests the energy storage is capacitance (*C*), which can be described by the following equation:

(1) $$C=\varepsilon_{0}\varepsilon_{r}A/d$$

where ε_0_ is the dielectric permittivity in vacuum (~8.85 × 10^−12^ F/m), ε*_r_* (>> 1) is the relative dielectric constant of the dielectric layer, A is the overlapping area of the electrodes, and *d* is thickness of the dielectric layer. In principle, the capacitance only depends on the geometry of the capacitor and permittivity of the dielectric layer. However, this is not always the case, especially when electric field-driven electrochemical activities take place near the dielectric/electrode interface, as discussed below.

When an external voltage is applied, charges with opposite signs and equal magnitude accumulate at the electrodes. This is the so-called charging process. The charges form an internal electric field, whose direction is opposite to that of the external electric field. The charging process finishes when the internal electric field induced by the accumulated charges (*Q = CV*) is equal to the external field (*E* = *V*/*d*), where *V* is the applied voltage. During the charging process, the charges are moved by the external electric field, and electrostatic energy is stored in the dielectric layer. The stored energy can be calculated from the following expression:

(2) $$W=\int_{0}^{Q_{max}}Vdq$$

where *Q*_max_ is the maximum charge when the charging process finishes, and *dq* is an increment of charge. One of the key figures of merit of the dielectric capacitor is its energy density (*J*), which measures its “capability” for storage performance and can be written in the following form:

(3) $$J=\frac{W}{Ad}=\int_{0}^{P_{max}}EdP$$

The energy density can therefore easily be obtained by integrating the area between the polarization and electric field axes in the *P-E* loop. Another key figure of merit for the capacitor is energy efficiency (*η*). It is equals to the ratio between the recoverable energy density (*J_rec_*) and total energy density (*J_tot_*), which can be expressed by

**Figure 2:**
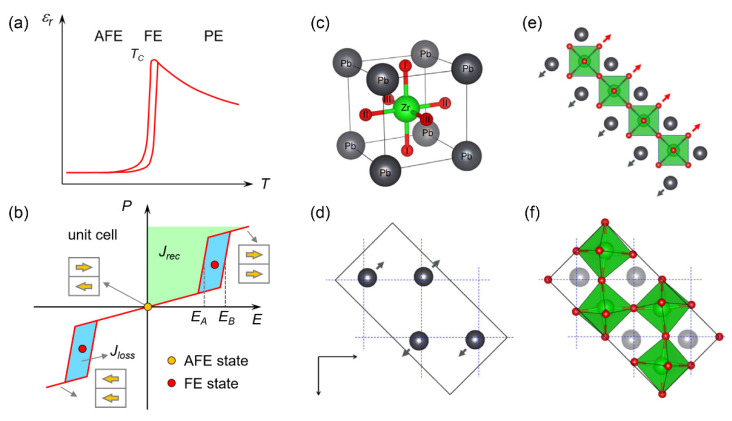
Dielectric constant (ε_r_), P-E loop and structural phases of PbZrO_3_. (**a,b**) Temperature-dependent ε_r_ and hysteresis loop of AFEs. The green and blue shaded areas in (**b**) denote recoverable (J_rec_) and loss (J_loss_) energy during the AFE-to-FE transition. (**c**) Unit cell of the cubic PbZrO_3_ phase (space group Pm$\bar{3}$m). (**d–f**) Antiparallel Pb displacements (Σ mode), polar displacement (Γ mode) and oxygen octahedral rotations (R mode) in AFE PbZrO_3_ (space group *Pbam*), respectively. The dotted lines show the cubic crystallographic axes in the ab plane [10].

*η* = (*J_rec_*/*J_tot_*) × 100% (4)

where the *J_tot_* is a sum of the *P-E* loop (*J_loss_*) and its left-side area (*J_rec_*) at *E* ≥ 0 in the *P-E* relation chart (see Figure 2b). It appears that a linear dielectric, with *J_rec_* ≈ $\frac{1}{2}\varepsilon_{0}\varepsilon_{r}E^{2}$ and very small *J_loss_*, may offer higher storage density. In practice, phase transition materials offer much higher energy storage densities due to their much larger *E_B_* and maximum polarization (*P_max_*), which can be one order of magnitude higher than that of linear dielectrics [39,55]. As for more circuit-related details of energy storage measurement, this has been discussed elsewhere [24,40].

From a microstructure point of view, complex structural changes take place during the charging process. Inside the nonpolar dielectric layer, this involves inversion symmetry breaking and the emergence of electric dipoles from the originally centrosymmetric lattices. Irrelevant to either displacive or order–disorder phase transition [56,57], the electric dipoles are defined by the separation of positive and negative charge centers within each unit cell. At mesoscopic scale, this is affected by phase constitution, domain reorientation and growth, defect type and density. Near the dielectric/electrode interface, polarization screening, chemical diffusion, ionic migration and potential M–I transition significantly impact the energy storage and release. Therefore, unveiling the dynamic structural responses under in situ conditions may greatly deepen our mechanistic understanding, which is essential for the development of new materials and devices.

## 3. Structural Features and Phase Transitions in Antiferroelectrics

FEs undergo a nonpolar-to-polar transition at *T_C_* and the breaking of spatial inversion symmetry leads to the emergence of spontaneous polarization (*P_S_*) in the low-temperature phase. Under the application of an electric field (*E*), the direction of polarization (*P*) in domains can be reversed and its nonlinear response to *E* gives rise to a *P*-*E* hysteresis loop. Depending on the category of the FEs, the hysteresis loop may vary between a square shape (e.g., in BaTiO_3_) [58] and a slim shape (e.g., in relaxor Na_0.5_Bi_0.5_TiO_3_) [37]. Thus, the energy consumption is usually larger for the former than the latter due to its larger *P_r_* (a value at *E* = 0) and loop areas. In contrast, AFEs undergo a phase transition between two nonpolar phases at *T_C_*, around which an FE phase may transiently exist in a narrow temperature window [59,60,61]; see Figure 2a. Despite zero spontaneous polarization at *T* < *T_C_*, a strong *E* can drive an AFE-to-FE phase transition [52,62], which gives rise to a double hysteresis loop; see Figure 2b. Due to the concave curvature of the *P-E* relationship, with a reflection point at coercive field *E_A_* or *E_B_*, the AFEs usually possess intrinsically high energy storage densities [63].

In a crystal structure, an important feature of AFE is that its unit cell volume is doubled with respect to its paraelectric (PE) phase (Figure 2c). Benefiting from the antiparallel arrangement of electric dipoles within sublattices, null polarization is obtained on the unit cell scale. Typical AFE systems include PbZrO_3_, AgNbO_3_, NaNbO_3_, HfO_2_ [64,65,66,67,68,69,70,71,72] and recently reported 2D van der Waals AFE *β*’-In_2_Se_3_ [73,74]. From the perspective of lattice dynamics, the emergence of antiferroelectricity is a consequence of competing lattice instabilities [10,11], which are manifested by delicate structural orders [66,75]. Taking PbZrO_3_ as an example, its AFE phase is dominated by antiparallel Pb displacements along the [100]_O_//[110]*_c_* direction (O: orthorhombic; *c*: pseudocubic) and antiferrodistortive (AFD) oxygen octahedra (*a^-^a^-^c^0^* in Glazer’s notation) [66]. The corresponding Σ and R modes in the Brillouin zone (Figure 2d,f) are represented by wave vectors of **k**_Σ_ = (2π/a)(1/4, 1/4, 0) (*a* is the cubic lattice constant) and **k**_R_ = (2π/a)(1/2, 1/2, 1/2), respectively [10,11].

A polar instability, i.e., the Γ mode, manifested by opposite shifts of Pb cations and oxygen anions, is responsible for ferroelectricity (Figure 2e). In the ground state, the polar instability is suppressed due to its higher energy level. Nevertheless, it has been shown that the energy levels of different lattice instabilities can be subverted in the following situations. (1) Application of large *E* can destabilize the Σ and R modes and drive an AFE-to-FE transformation [76,77,78]. (2) Interruption of the cationic displacement order and AFD order at translational boundaries allows the emergence of local ferroelectricity with a bi-stable feature [79,80,81]. (3) An intrinsic surface effect may trigger an AFE-to-FE transition as the sample thickness is below a critical thickness, ~6.5 nm for PbZrO_3_ [82,83]. (4) Chemical doping can also alter the cationic displacement order and AFD order, e.g., stochastic stacking of Pb-displacement-based stripes in La-doped PbZrO_3_. Due to the uncompensated antiparallel arrangement of Pb cations, weak ferrielectricity emerges in doped systems [84,85,86].

## 4. Antiferrodistortive and Ferrodistortive Phase Transitions

**Figure 3:**
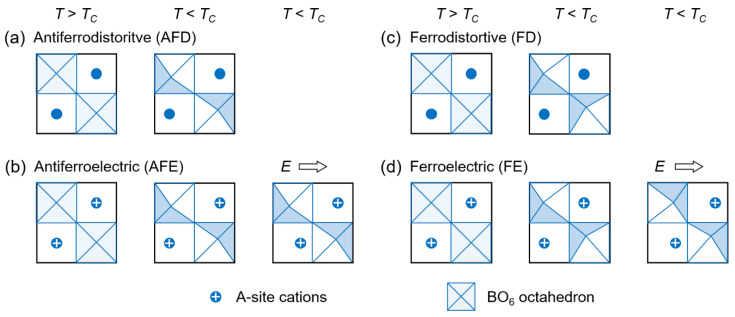
Structural phase transitions of a centrosymmetric perovskite oxide (ABO_3_). (**a,c**) Antiferrodistortive (AFD) and ferrodistortive (FD) transitions caused by temperature or stress, respectively. Out-of-phase or in-phase octahedral rotation is linked in (**a**) but can be unlinked in (**c**) due to polar distortion. (**b,d**) Antiferroelectric (AFE) and ferroelectric (FE) transitions caused by temperature and electric field *E*, respectively. Associated with symmetry lowering, polar octahedra in an FD phase may respond to applied electric field together with A-site cations.

In fact, both FE-to-PE and AFE-to-PE transitions can be understood from the viewpoint of ferroelastic phase transition, which is related to a change in lattice symmetry around *T_C_* [87,88]. In many cases, ferroelasticity or antiferroelasticity is a secondary or improper effect, since the driving force is related to cation or molecular ordering and the softening of optical phonon branches [89,90]. Thus, a large proportion of ferroelastic transitions depend on mode condensation at a position in the Brillouin zone. When modes condense at zone boundaries, the transition is termed AFD [91]. For a perovskite, the AFD order is manifested by in-phase and out-of-phase rotation of the oxygen octahedra [92,93]. Thus, the AFEs constitute a subgroup of AFD transitions (Figure 3a,b). In contrast, when modes condense at the zone center, the transition is termed FD. Correspondingly, the FEs constitute a subgroup of FD transitions (Figure 3c,d). It should be noted that the AFD phases are characterized by the rigid octahedral linking through out-of-phase tilting, in-phase tilting or their combination. In FD phases, associated with symmetry lowering, polar octahedral distortion can modify or destroy the rigid linking and response to the applied electric field together with A-site cations. In addition, there are also antipolar (AP) and pyroelectric (PyE) phases, which belong to the subgroups of AFD and FD transitions, respectively. In contrast to AFE and FE phases, their dipoles cannot be reversed, even by a strong electric field [91].

It should be noted that the zone-center mode condensation is not necessarily bound to the zone-boundary condensation in proper FEs. This means that the polar order can either be irrelevant to the AFD order (e.g., in BaTiO_3_ and PbTiO_3_) or couple cooperatively with the AFD order (e.g., in BiFeO_3_, ZnSnO_3_ and ScFeO_3_) [94,95,96]. Besides the routine manifestations, as a counterpart of AFD, the FD phase transition may place extra emphasis on a means of inducing ferroelectricity through the long-range polar distortion of oxygen octahedra. This broadens the perspectives of understanding the origins of ferroelectricity and implies that more nonpolar dielectrics with typical AFD order [92,93] can be transformed to novel FD phases under excitation of an external field [97]. Being compatible with chemical, defect and strain engineering [30,98], therefore, the range of candidate energy storage systems can be greatly expanded by the AFD-to-FD transition. 

**Figure 4:**
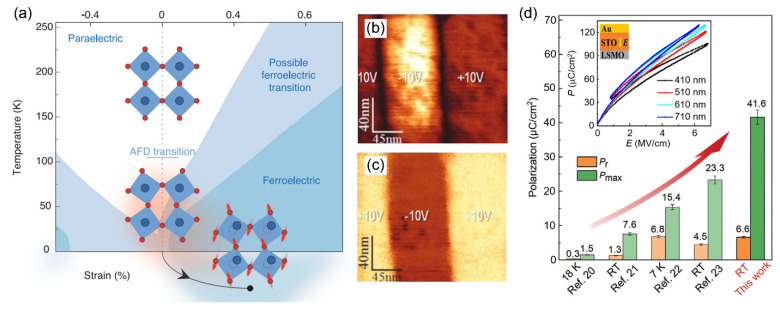
Structural phase transition and FE properties of SrTiO_3_. (**a**) Temperature–strain phase diagram showing an AFD transition and a further FE transition [102]. (**b,c**) Piezoresponse force microscopy (PFM) amplitude and phase images of strain-free SrTiO_3_ films recorded at 50 K at biasing voltages of ±10 V [29]. (**d**) Comparisons of *P_r_* and maximum polarization (*P_max_*) between Ti/O-deficient SrTiO_3_ (*P_r_* = 6.6 μC/cm^2^, *P_max_* = 41.6 μC/cm^2^) and the literature cited therein [30]. The inset shows the *P-E* loops measured from the Au/SrTiO_3_/LSMO (x ≈ 1/3) capacitors [28].

With reference to the above classifications, the STO/LSMO system, with ultrahigh energy density (307 J/cm^3^) at a thickness range of 410~710 nm for STO, provides a good example to analyze the AFD-to-FD transition [28,99]. It has been widely accepted that the cubic SrTiO_3_ (space group *Pm*$\bar{3}$*m*) transforms to a tetragonal structure (*I*4/*mcm*) via a second-order AFD phase transition at *T_C_* = 105 K. Below *T_C_*, the tetragonal phase is characterized by the antiphase rotation of the octahedra along its *c* axis (*a^0^a^0^c^-^* in Glazer’s notation) [100]. The temperature–strain phase diagram [101,102,103,104] shows that the FE phase can be stabilized through strain engineering (Figure 4a). Nonetheless, the following results indicate that the mechanism leading to polarity in SrTiO_3_ is not that simple. Compared with strained SrTiO_3_, Jang et al. demonstrate that strain-free SrTiO_3_ is an RFE, with a temperature for the maximum *ε_r_* at *T_m_* ≈ 45 K [29]. The role of strain is to stabilize the long-range correlation of preexisting nanopolar regions; see Figure 4b,c. Another argument points out that the abnormal ferroelectricity at *T* < *T_m_* arises from FE antiphase boundaries [105,106,107]. Similar to AFE PbZrO_3_, once the nonpolar lattice instabilities become destabilized, the “hidden” polar lattice instability prevails and gives rise to the ferroelectricity.

Excited by either an optical pump or a THz electric field (with a threshold field amplitude of ~300 kV/cm), two research groups independently reported that metastable ferroelectricity with *T_C_* ≈ 290 K can be achieved in SrTiO_3_ [102,108]. By introducing Ti/O-deficient nanoregions [30], Li et al. showed that defect engineering can also drive the occurrence of AFD-to-FD transition. Using this strategy, they realized a very large lattice tetragonality ratio (*c*/*a* = 1.038), strong room-temperature ferroelectricity (*P_S_* = 41.6 μC/cm^2^) and very high *T_C_* (~1098 K) in SrTiO_3_ films; see Figure 4d. Using first-principles calculations, Klyukin and Alexandrov reported that antisite defects, either Ti on a Sr site (Ti_Sr_) or vice versa (Sr_Ti_), can result in large electric polarization in SrTiO_3_ [109,110,111]. These outcomes suggest that structural defects should play an important role in achieving ultrahigh polarization in the STO/LSMO system (see inset of Figure 4d). More importantly, this sets a precedent for retrofitting other nonpolar perovskites into energy storage media via chemical and defect engineering, e.g., CaTiO_3_ and DyScO_3_ [55,98,112].

## 5. Anisotropic Energy Storage 

**Figure 5:**
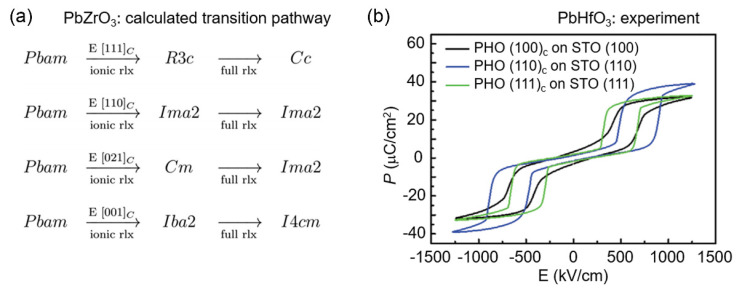
Anisotropic energy storage pathways. (**a**) Predicted phase transition pathways for PbZrO_3_ associated with ionic and full structural relaxation after applying electric fields along different crystallographic directions. The multiscale structure prediction combines classical and first-principles density functional theory [114]. (**b**) Structural anisotropy-dependent energy storage performance of AFE PbHfO_3_ thin films grown on different crystal-plane-terminated SrTiO_3_ substrates [71].

The double *P-E* hysteresis loop shown in Figure 2b indicates that synergistically changing the following parameters may optimize the energy storage performance: (1) simultaneously increasing the critical electric fields *E_A_* and *E_B_* and minimizing the loop area [31]; (2) increasing the *P_max_* [28] and minimizing the *P_r_* [32]. In practice, due to the dielectric anisotropy of the crystals [113], the energy storage density of identical materials also exhibits a direction-dependent nature [71,97,114,115]. Taking PbZrO_3_ as an example, multiscale first-principles computations show that as the *E* is applied along different crystallographic directions, its phase transition pathways are distinct (Figure 5a). On this basis, Lisenkov et al. found three high-strain polar phases: a monoclinic (*Cc*) phase, an orthorhombic (*Ima2*) phase and a tetragonal (*I4cm*) phase [114].

In experiments, the key electrical parameters relating to energy storage also show strong crystal orientation dependence. AFE (200 nm) PbHfO_3_ films grown on (100)_C_-plane, (110)_C_-plane and (111)_C_-plane terminated SrTiO_3_ substrates, which are buffered by SrRuO_3_ electrodes, are good examples that confirm this [71,72]. Together with large changes in critical field and *P_r_*, the *P_max_* takes values of 30.54, 38.91 and 32.64 μC/cm^2^ in the three films, respectively (Figure 5b). Corresponding to a larger coercive field and *P_max_*, the (110)_C_ oriented film delivers higher energy storage density (*J_rec_* = 21.4 J/cm^3^) than the other two (*J_rec_* ≈ 16 J/cm^3^) [71]. From a microstructural perspective, one may expect that the type and density of structural defects [116,117,118,119] may influence the energy storage performance. Through introducing local compressive pressure, Zhang et al. reported that enhanced critical fields may increase the *J_rec_* [53,67,120], e.g., from 9 to 16.2 J/cm^3^ in Li^+^-La^3+^ co-doped PbZrO_3_ films. By constructing a ferrielectric (FiE) M2-M3 phase boundary, Luo et al. achieved an energy density of 6.3 J/cm^3^ with *η* = 90% in (1-x)AgNbO_3_-xAgTaO_3_ solid solution [46].

## 6. In Situ Structural Shase Sransition Sathway

In conjunction with macroscopic property measurement, in situ dynamic structural studies can help to construct structure–property relationships directly. Under the application of an electric field, answers to the following questions are expected to play critical roles in further improving the energy storage performance. Q1) How does electrical polarization emerge and evolve from a nonpolar lattice matrix? Q2) What are the key structural factors that control and limit critical fields (*E_A_* and *E_B_*), *P_max_* and *P_r_*? Although the phase transition processes are complex, as reported in PbZrO_3_-based and NaNbO_3_-based systems [121,122,123,124], manipulative free dimensions about spatial, temporal and electric field offer a plethora of opportunities to unveil the unknowns. For example, in Pb(Zr_0.57_Sn_0.43_)O_3_-based ceramics, Fan et al. observed local depolarization field-assisted AFE-to-FE transition during monotonic *E* loading using in situ (scanning) transmission electron microscopy (S/TEM). In addition to this, they also found suppression of FE domain mobility after 10^3^-time bipolar cycling, which indicates the electric fatigue of the FE phase [125].

**Figure 6:**
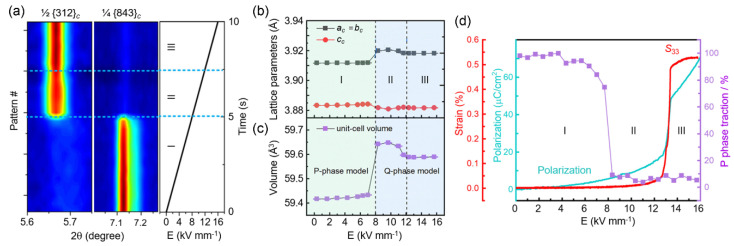
Time-dependent phase transition in NaNbO_3_ under excitation of electric field. (**a**) Evolution of XRD superlattice reflections with increasing electric field for the AFE P (stage I) and FE Q (stage III) phases. (**b,c**) Pseudocubic lattice parameters and unit cell volume plotted as a function of electric field amplitude. (**d**) Evolution of the P-phase fraction, macroscopic polarization and longitudinal strain *S*_33_ as a function of electric field [126]. Instead of the decoupled polarization switching process, stage II may also be interpreted as the emergence of an intermediate FiE phase.

By largely improving the temporal resolution under in situ biasing conditions, from ~30 s to 415 ms per data pattern, Zhang et al. investigated the dynamic structure evolution of polycrystalline NaNbO_3_ using high-energy XRD (Figure 6a). Associated with the disappearance and appearance of specific superlattice reflections, e.g., ½(312)*_c_* reflection, they found that an AFE-to-FE transition took place around *E* = 8 kV/mm [126]. At *E* > 12 kV/mm, abruptly enhanced polarization and longitudinal strain *S*_33_ indicated the transformation of the nonpolar P phase (orthorhombic AFE, *Pbcm*) to the polar Q phase (orthorhombic FE, *P2_1_ma*) (Figure 6b–d). According to the mismatch of the P-phase fraction with changes in lattice parameter, volume, polarization and *S*_33_, they proposed a decoupled polarization switching process in the range of 8 < *E* < 12 kV/mm. Given that the P phase (*a^-^a^-^b^+^*/*a^-^a^-^b^-^*/*a^-^a^-^b^+^*) differs from the Q phase (*a^-^a^-^b^+^*) in octahedral tilting, the field-dependent structural data cannot exclude another possibility, i.e., the existence of an intermediate FiE phase. It adopts a Q-phase structural framework [98], but the oxygen octahedra are in a transitional state. Observation of weak ferrielectricity in PbZrO_3_-based ceramics supports this possible interpretation [85,86,127].

Compared with diffraction-based techniques, *E*-dependent structural transition, measured at atomic scale using S/TEM, provides more intuitive information about energy storage. However, even using a state-of-the-art imaging detector such as the K3 camera, which is capable of collecting 1500 data frames per second (0.67 ms per frame), the mismatch in time remains due to the requirement of sufficient signal intensity and image contrast for data analysis. Thus far, several advanced imaging techniques have been used to image light elements, e.g., negative spherical aberration imaging (NCSI) [128,129,130], annular bright field (ABF) imaging [131], integrated differential phase contrast (iDPC) imaging [132,133] and electron ptychography [134]. Despite this, imaging light elements such as oxygen under in situ biasing conditions remains a challenge due to their very low scattering power. As a compromise, slowing down the phase transition speed becomes a good solution to unravel the evolution of characteristic structural orders as a function of field strength and time [52].

**Figure 7:**
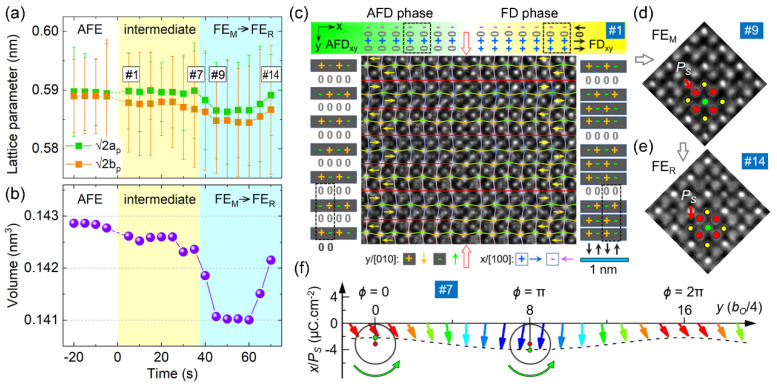
Structural phase transitions of PbZrO_3_ induced by electron-beam irradiation in a TEM. (**a,b**) Changes in lattice parameter (√2*a*_p_, √2*b*_p_) and volume (*V* = √2*a*_p_ × √2*b*_p_ × *c*_p_) plotted as a function of irradiation time, respectively. (**c–e**) Atomic-resolution NCSI-TEM images of an AFD-FD phase boundary and associated structural phase transitions recorded along [001]_O_ direction, respectively. The oxygen displacements in (**c**) are indexed by (+, 0, –) symbols and colorful solid lines. The yellow arrows denote antiparallel Pb displacements. The atom types are Pb—yellow, Zr—green, O—red circles, respectively. (**f**) 2D cycloidal order of polarization for the transient FE-FD phase obtained using quantitative TEM study [127].

Recently, Wei et al. found that an illumination electron beam in a TEM can act as an external electric field to trigger phase transitions in dielectric insulators. The principle lies in the fact that the electrons captured by the insulating sample surface are “equivalent” to the electrostatic charging effect [135,136]. By slowing down the phase transition speed to the level of seconds, the investigation of atomic-scale structural changes becomes possible [127,137,138]. For a [001]_O_-oriented PbZrO_3_ lamella sample, time- and atomic-resolution NCSI-TEM study [127] reveals that the AFE-to-FE transition involves the splitting of pseudocubic *a_p_* and *b_p_* axes. As a function of irradiation time, the unit cell volume undergoes a reduction-to-expansion transition as the AFE phase evolves into FE monoclinic (FE_M_) and rhombohedral (FE_R_) phases (Figure 7a–e). During the in situ energy storage process, an intermediate transient FD phase was observed between the AFE and FE phases. With the preservation of antiparallel Pb displacements, polar octahedral distortion takes place and breaks the spatial inversion symmetry along the x direction. Associated with the extraction of atomic positions through quantitative TEM study, the authors found that the transient FD phase exhibits a cycloidal order of polarization, with *P_S_* ≈ 2.8 μC/cm^2^ (Figure 7c,f). The finding of this FiE phase suggests the origin of the linear polarization response at *E* < *E_A_*_(*B*)_ in the *P-E* loop of PbZrO_3_. Synchrotron X-ray and neutron diffraction structural studies reported similar FiE behavior, characteristic of a wavy polarization order, in ternary PbZrO_3_-PbSnO_3_-PbTiO_3_ solid solutions [85,86,139].

**Figure 8:**
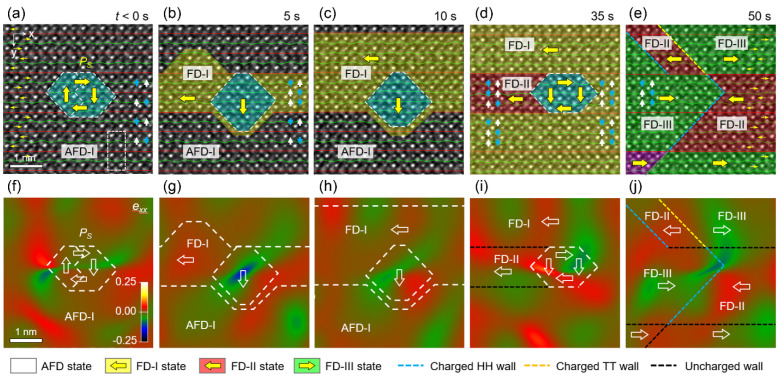
Defect-core-induced unit-cell-wise energy storage pathway in [001]_O_-oriented PbZrO_3_. (**a–e**) Phase and domain structure evolution as a function of electron-beam irradiation time (dose rate = 3.8 × 10^6^ e/nm^2^⋅s). The cyan shadow and thick yellow arrows denote the defect core and polarization, respectively. (**f–j**) Corresponding lattice strain *e_xx_* (horizontal [100]_O_ direction) maps obtained using geometric phase analysis (GPA) [118].

Regarding the phase transition pathway, we should note the possible difference between calculated models and experimental observations (Figure 5 and Figure 7). For the latter, structural defects such as atomic vacancies and stoichiometry issues prevail in real samples [140,141], and they may potentially influence the transition pathway. By using time- and atomic-resolution NCSI-TEM, Wei et al. found that a defect core, induced by oxygen and Pb vacancies [118], can act as a seed to trigger unit-cell-wise AFD-to-FD transition in [001]_O_-oriented PbZrO_3_ (Figure 8a–e). This is in sharp contrast to the case with the absence of such a defect core, where a relatively uniform AFD-to-FD transition takes place in the region irradiated by the electron beam [127]. As a result of the seed effect, charged FD domains with head-to-head and tail-to-tail configurations are observed during the energy storage process.

Compared with neutral domain walls, we know that the formation energy of charged domain walls is much higher [117,142] and 2D electron gas may form at the walls to compensate for bound charges and lower the electrostatic energy [113,143]. The anisotropic structural transformation suggests that the defect cores probably lower the coercive field (*E_B_*). With the preservation of antiparallel Pb displacements, this further proves that the energy storage and transfer begin with distorting the oxygen octahedral network. Another point worth noting is the evolution of the polar configuration inside the defect core. At the initial AFE state, electric dipoles form a self-compensated vortex structure. Accompanied by energy injection, the local polarity is stabilized until immersion in the FD states. Strain analysis reveals that nanoscale local compressive strain leads to the seed effect of the defect core (Figure 8f–j). This points out that the rational selection of the doping element and concentration is crucial for optimizing the storage performance of dielectrics [144,145,146].

## 7. Ionic Migration across Dielectric/Metal Interfaces

In oxide-based dielectric capacitors, metallic (La_1−x_Sr_x_)MnO_3_ [147], (La_1−x_Sr_x_)CoO_3_ [148] and SrRuO_3_ [149] are standard choices for bottom electrodes. Previous transport property studies have reported that (La_1−x_Sr_x_)MnO_3_ undergoes thickness-driven and electric-field-driven M–I transition [137,150,151]. When such a transition occurs in dielectric capacitors, “dead” layers appear at the dielectric/electrode interfaces and this may impact the energy storage performance. Driven by the depolarization effect, the “dead” layers usually lead to reduced capacitance near the interface [152,153,154]. The contrary phenomenon observed in the STO/LSMO system (*P_S_* ≈ 125 μC/cm^2^) suggests that the interface should contribute positively to the “defect-induced” ferroelectricity in SrTiO_3_ (Figure 4d). Hou et al. ascribe the enhanced *E_B_* to the modulation of the local electric field and redistribution of oxygen vacancies at the oxide interface [28]. However, the finite interface thickness (~5 nm), compared to that of ~560 nm for SrTiO_3_, casts doubt on the role of the interfacial contribution alone.

**Figure 9:**
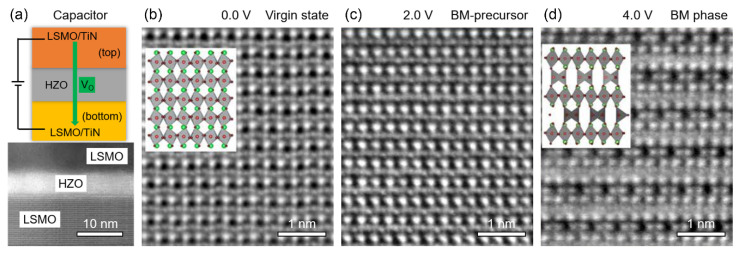
Deoxygenation of the bottom (La_1-x_Sr_x_)MnO_3_ (LSMO, x ≈ 1/3) electrode with increasing positive bias in an LSMO/HZO/LSMO capacitor. (**a**) Polarity-dependent oxygen voltammetry process (V_O_: oxygen vacancy) and morphology of the dielectric capacitor. (**b–d**) The iDPC-STEM images of a representative region of the bottom LSMO layer, showing (**b**) the virgin 0.0 V state, (**c**) the BM precursor phase (with mixed MnO_5_ and MnO_6_ polyhedra) at 2.0 V and (**d**) the BM phase at 4.0 V viewed along the [110]*_c_* zone axis. The atom types in the insets are La/Sr—green, Mn—red and O—brown [158].

Rhombohedral LSMO (x ≈ 1/3, space group *R*$\bar{3}$*c*) has a ferromagnetic metallic state at room temperature. Below *T_C_* ≈ 367 K, its structure is characterized by AFD octahedral rotations (*a^-^a^-^a^-^* in Glazer’s notation) [147,155]. By using in situ biasing STEM, Yao et al. revealed an *E*-induced M–I transition by measuring the *E-*dependent resistivity change. The insulating state was attributed to the formation of a brownmillerite (BM) phase (*C2/c*), which has a bandgap of *E_g_* ≈ 0.63 eV [151,156,157]. In Hf_0.5_Zr_0.5_O_2_ (HZO, 5 nm)/LSMO films (Figure 9a), Nukala et al. directly observed oxygen vacancy-induced structural transition in bottom LSMO by using in situ biasing iDPC STEM [158]. In addition to an intermediate BM precursor phase, they found the intertwining of polarization switching with the migration of oxygen vacancies (Figure 9b–d). Specifically, the role of the dielectric oxide layer, either as a fast conduit or as a source/sink of oxygen migration depending on the oxygen reactivity of the top electrode [158,159], highlights the necessity of considering electrochemical activities for capacitors under working conditions. This may involve the migration of atomic vacancies and cations through antisite defects, and electronic structure changes in all atomic species. The ultrahigh energy density achieved in Na-based perovskite oxides, e.g., ionic conductor (Na_0.5_Bi_0.5_)TiO_3_ [160], also suggests the important role of ionic migration in enhancing the energy storage performance; see Table 1. In addition, possible FE metal states [161,162,163] should be considered at the dielectric/metal interfaces. These mechanisms may apply to the nonpolar Au/STO/LSMO and other systems [164,165].

## 8. Summary and Outlook 

By generalizing the AFE-to-FE transition to a broader AFD-to-FD transition, we summarize in situ phase transition pathway studies in energy storage nonpolar materials. Aiming at identifying the underlying mechanism and improving the energy storage performance of dielectric capacitors, we highlight several key points below to inspire future research.

(I) Besides mesoscale domain structure and phase boundary evolution, light element sensitive imaging techniques should play a key role in unraveling the in situ dynamic phase transition during energy storage. The reason lies in the fact that the delicate energy transfer usually starts from light-element-based structural units, e.g., distortion of the oxygen octahedra and migration of oxygen vacancies. Specifically, transient FE or FiE phases may possibly emerge under non-equilibrium conditions. Apart from deepening our understanding of the energy storage process, such studies promise to expand the recognition scope of FE physics.

(II) Case studies of PbZrO_3_, SrTiO_3_ and (La_1−x_Sr_x_)MnO_3_ highlight the significance of carrying out defect and interface structure investigation during the energy storage processes. This may include dopant-induced strain change, antisite mechanism and phase boundary evolution under in situ biasing conditions. Establishing the direct microstructure–property relationship may help to optimize the figures of merit of the *P-E* loops, e.g., increasing the *P_max_* and critical fields while reducing *P_r_* and the loop area.

Finally, two recent works reported that mesoscale-domain engineering may greatly improve the energy storage density, e.g., from 12.2 to 18.5 J/cm^3^ in NaNbO_3_-based relaxor AFE ceramics [44,166]. Pertinent to the beneficial hierarchical domain structures, structure–property relationship study of FE Pb(Zr,Ti)O_3_ shows that this may arise from phase transition frustration near a tricritical point [167]. Together with the use of advanced computation and simulation methods, such as machine learning, we believe that carrying out cross-scale microstructure study may boost the development of energy storage materials and their device application.

## Figures and Tables

**Table 1:** Comparison of energy storage performance between representative dielectric films and between representative dielectric bulks.

**Dielectric Films**	***J_rec_* (J/cm^3^)**	***η* (%)**	***P_max_* (** **μC/cm^2^)**	***E_B_* (MV/cm)**	**Ref.**
SrTiO_3_^(1)^	307	89	~125	6.8	[28]
La-Ba-Zr-doped (Na_0.5_Bi_0.5_)TiO_3_^(1)^	154	97	113.5	3.5	[37]
Sm-doped BiFeO_3_-BaTiO_3_^(2)^	152	> 90	~60	5.2	[38]
BaZr_0.35_Ti_0.65_O_3_ multilayer^(2)^	130	73.8	52	8.75	[41]
Mn-doped Na_0.5_Bi_0.5_TiO_3_-BaTiO_3_-BiFeO_3_^(3)^	102	60	124	2.86	[42]
La-doped Pb(Zr,Ti)O_3_^(4)^	85	65	115	4.5	[43]
Pb_0.5_Sr_0.5_HfO_3_^(5)^	77	97	~53	5.12	[39]
**Dielectric bulks**	***J_rec_* (J/cm^3^)**	***η* (%)**	***P_max_* (** **μC/cm^2^)**	***E_B_* (MV/cm)**	**Ref.**
Na_0.5_Bi_0.5_TiO_3_-Sr_0.7_Bi_0.2_TiO_3_^(3)^	21.5	~80	67	103	[36]
0.90NaNbO_3_-0.10BiFeO_3_^(6)^	18.5	78.7	64	~1.0	[44]
La-doped Pb(Zr_0.55_Sn_0.45_)_0.995_O_3_^(7)^	10.4	87	41.3	0.4	[32]
(Pb_0.91_Ba_0.045_La_0.03_)(Zr_0.6_Sn_0.4_)O_3_^(7)^	8.16	92.1	40	0.34	[31]
BiFeO_3_-BaTiO_3_-NaNbO_3_^(6)^	8.12	90	~52	0.36	[45]
AgNbO_3_-AgTaO_3_^(6)^	7.5	86	32	0.53	[46]

Note: The bottom electrode types are (1) La_0.67_Sr_0.33_MnO_3_, (2) Nb-SrTiO_3_, (3) Pt, (4) LaNiO_3_, (5) SrRuO_3_, (6) silver paste, (7) Au.

## Data Availability

Not applicable.
